# A Cross-Sectional Study Examining Differences in Indication for Cesarean Delivery by Race/Ethnicity

**DOI:** 10.3390/healthcare9020159

**Published:** 2021-02-03

**Authors:** Rebecca Delafield, Jennifer Elia, Ann Chang, Bliss Kaneshiro, Tetine Sentell, Catherine M. Pirkle

**Affiliations:** 1 Thompson School of Social Work & Public Health, Office of Public Health Studies, University of Hawai‘i at Mānoa, Honolulu, HI 96822, USA; tsentell@hawaii.edu (T.S.); cmpirkle@hawaii.edu (C.M.P.); 2 John A. Burns School of Medicine, Department of Obstetrics, Gynecology and Women’s Health, University of Hawai‘i, Honolulu, HI 96826, USA; jennifer@ecashawaii.org (J.E.); annchang@hawaii.edu (A.C.); blissk@hawaii.edu (B.K.)

**Keywords:** cesarean section, decision making, indications for cesarean delivery, Micronesia, non-reassuring fetal heart tracing, provider bias, healthcare disparities, Hawai‘i

## Abstract

(1) Background: There are persistent racial/ethnic disparities in cesarean delivery in the United States (U.S.), yet the causes remain unknown. One factor could be provider bias. We examined medical indications for cesarean delivery that involve a greater degree of physician discretion (more subjective) versus medical indications that involve less physician discretion (more objective) to better understand factors contributing to the higher rate among Micronesian, one of the most recent migrant groups in the state, compared to White women in Hawai‘i. (2) Methods: A retrospective chart review was conducted to collect data on 620 cesarean deliveries (N = 296 White and N = 324 Micronesian) at the state’s largest maternity hospital. Multivariate regression models were used to examine associations between maternal and obstetric characteristics and (1) subjective indication defined as non-reassuring fetal heart tracing (NRFHT) and arrest of labor disorders, and (2) objective indication defined as all other indications (e.g., malpresentation). (3) Results: We found that Micronesian women had significantly higher odds of cesarean delivery due to a subjective indication compared to White women (aOR: 4.17; CI: 2.52-6.88; *P* < 0.001; N = 619) after adjusting for multiple covariates. (4) Conclusion: These findings suggest unmeasured factors, possibly provider bias, may influence cesarean delivery recommendations for Micronesian women in Hawai‘i.

## 1. Introduction

In 2003, the Institute of Medicine summarized evidence of racial/ethnic disparities in the United States (U.S.) and concluded that stereotyping and provider bias contribute to inequities in health [1]. Persistently higher rates of cesarean delivery in certain racial/ethnic groups in the U.S. and other nations remain largely unexplained by medical and socio-demographic risk factors, raising questions about underlying bias in obstetric care [2,3,4,5,6]. Decision-making with regard to cesarean delivery is complex and involves the patient, the provider, and both objective and subjective assessments, which can be an ingress for conscious or unconscious biases to influence decisions. Both the American College of Obstetricians and Gynecologists and the Society of Maternal and Fetal Medicine have highlighted the need for additional research into implicit biases within the field of obstetrics and gynecology [7,8].

Medical indications for cesarean delivery can differ in the level of clinician discretion required to assess them. For example, there is noted variation in interpretation of fetal heart tracings between clinicians [9,10]. Non-reassuring fetal heart tracing (NRFHT) and arrest of labor disorders are categorized as more subjective indications for cesarean delivery when compared to malpresentation or obstetric factors [11]. While disparities in cesarean delivery are noted, there is relatively little research examining variation in indications for cesarean delivery by race/ethnicity, leaving substantial knowledge gaps regarding the mechanisms that may contribute to differences in rates [12]. Of the limited studies that have examined cesarean delivery indications by race/ethnicity, findings indicate that Black women are more likely than White women to have cesarean delivery for NRFHT, but the reason for this difference is unclear [2,13].

Here, we examine medical indications for cesarean delivery in a Pacific Islander population—Micronesian women—previously shown to have significantly higher cesarean delivery rates than Whites (32.3% vs. 26.8%) in Hawai‘i [14], where the overall rate is 25.6% [15]. Hawai‘i is a state that is highly racially/ethnically diverse where no single racial/ethnic group is in the majority. Pacific Islanders (not including Native Hawaiians) make up 4% of the population, and 25% of the population identify as White. Generally, there is a dearth of data on maternal health outcomes for Pacific Islanders in the U.S. despite the fact that they represent one of the fastest growing racial/ethnic populations in the country. Groups affiliated with island communities in Micronesia, a vast region in the western Pacific Ocean, had the largest percent increases in population compared to other Pacific Islander groups in the U.S. [16]. In Hawai‘i, a substantial and growing proportion of people from Micronesia trace their roots to the island nation states of the Federated States of Micronesia (FSM), the Republic of the Marshall Islands (RMI), and, to a lesser extent, the Republic of Palau (ROP) [16]. These communities are distinct in terms of ethnic identities and languages (e.g., Chuukese, Kosrean, Palauan and Marshallese), but are bound in important ways by their unique political affiliation with the U.S. and the historic and contemporary impacts of U.S. interest and military involvement in the region. Under Compact of Free Association (COFA) agreements, citizens of the FSM, RMI, and ROP are allowed visa-free entry into the U.S. and can legally live and work in the country in exchange for granting the U.S. exclusive military control of the islands [17]. Since the first COFA agreements were signed in 1986, migration to the U.S. has increased dramatically. Many families moved to the U.S. for healthcare services, educational opportunities, and jobs unavailable in their islands [15]. Unfortunately, similar to other communities of color in the U.S., Micronesians in Hawai‘i have faced prejudice and discrimination in multiple sectors, including in healthcare [18,19,20].

Considering that previous research found Micronesian women have a higher likelihood of cesarean delivery compared to White women after adjusting for diabetes, hypertension, and a number of other risk factors, a closer look at the indications driving this difference in rates is warranted. By comparing indications for cesarean delivery between White and Micronesian women, an approach largely absent from previous work on the topic, our aim was to better understand factors contributing to racial/ethnic disparities in this procedure. Our study hypothesis was that Micronesian women are more likely to have a subjective indication (e.g., NRFHT) for cesarean delivery compared to White women.

## 2. Materials and Methods

We conducted an observational cross-sectional study using retrospective medical chart review from a large not-for-profit maternity hospital in Hawai‘i, USA. The hospital serves as a teaching hospital and provides labor and delivery care for patients with private or public insurance. Approximately 6000 of the estimated 13,500 births that occur annually on the island of O‘ahu, where nearly 70% of the state’s population resides and where most high-risk pregnancies from other islands receive care, are served by this single institution [21,22]. This study was reviewed and approved as exempt by the University of Hawai‘i Institutional Review Board on 13 September 2017 (CHS#2017-00567).

We received data on 4476 cesarean deliveries (identified by ICD-9 codes) that occurred at the maternity hospital between 1 January 2010 and 31 December 2016. Cesarean deliveries eligible for analysis were those performed on Micronesian or White women during this time period. Race/ethnicity was derived from birth certificate data. The Micronesian group was defined by geographic region (consistent with existing maternal health literature) and included individuals who reported only a single Micronesian race/ethnicity or mix of Micronesian races/ethnicities (Micronesian, Chamorro, Guamanian, Chuukese, Marshallese, or other Micronesian group). Race/ethnicity was coded White only when listed alone or if the other ethnicities listed also were categorized as Caucasian by the U.S. Office of Management and Budget [23].

A total of 2182 cesarean deliveries met the eligibility criteria. Because of the time required to complete the manual extraction (described below), we could not analyze all 2182 deliveries. Random number generation was thus used to randomly select 620 records of cesarean deliveries (324 Micronesian and 296 White women) for analysis, representing 52% and 48% (respectively) of the full sample for each race/ethnic group. A sample of 600 (based on an α of 0.05 and β of 0.20) was calculated as necessary to detect a difference of 10% in the proportion of those receiving a cesarean delivery for a given indication.

Study data were collected from the electronic medical record (EMR) through automated extraction and manual chart review. Not all the data for this study could be easily pulled from the electronic record for efficient use in a database. For example, unless a patient’s prenatal care was provided by an OB-GYN with the same EMR platform used at the hospital, prenatal records were added to the EMR as scanned files and unable to be pulled electronically. Therefore, a manual chart review was conducted to collect data on variables that we were unable to extract electronically (e.g., initial prenatal visit) and as a check on the quality of data electronically extracted (e.g., parity). For the manual abstraction, a study-specific data abstraction tool was developed to collect data from several sources in the EMR, including the delivery, prenatal, and admission records. The tool was reviewed by experts (physicians that use the databases and researchers with experience doing retrospective chart reviews) to solicit feedback. After, it was pre-tested with a convenience sample of approximately 15 records to ensure ease of use. Refinements addressed issues identified in the initial test.

The primary outcome for the study was the indication for cesarean delivery as written in the operative note in the hospital’s EMR. An obstetrician on the study team (A.C.) abstracted the indication for cesarean delivery for all 620 study records. Indications for cesarean delivery were then recoded into related categories of indications guided by previous research on the topic [2,11]. Categories included: (1) arrest of labor (e.g., arrest of dilation, arrest of descent, cephalopelvic disproportion, failed induction), (2) NRFHT, (3) malpresentation (e.g., breech, transverse lie), (4) repeat cesarean, (5) multiple gestation, (6) obstetric conditions (e.g., cord prolapse, placenta previa), (7) maternal conditions (e.g., active herpes simplex virus, pre-eclampsia, vaginal septum), (8) fetal conditions (e.g., intrauterine growth restriction, fetal anomalies), and (9) other (i.e., any other indication that is not specified above). Because of the small number of maternal (N = 11), fetal (N = 17), and obstetric (N = 19) conditions, these indications were grouped together under the “other” category, reducing the total number of categories to six. While cesarean delivery on maternal request absent of a medical indication may be an issue in some contexts, it is relatively limited (about 2.5% of births) in the U.S. [24] and was not noted as an indication in any of the records reviewed for this study [25,26]. Indications were also recoded into a dichotomous variable of subjective (NRFHT and arrest of labor) and objective (all other indications). This categorization was based on previous work by Morris et al. (2016) and others highlighting the greater physician discretion required to diagnose NRFHT and arrest of labor as opposed to other indications, e.g., breech, placenta previa, pre-eclampsia [2,3,11,27].

The primary exposure variable of interest in this study was race/ethnicity, specifically Micronesian compared to White. A number of demographic, provider, risk factor, and obstetrical variables were collected. Variable selection was based on previous research relating many of these factors to increased or decreased risk of cesarean delivery [3,14]. With the exception of indication for cesarean, manual abstraction of data from the EMR was conducted by two study team members (R.D. and J.E.) following protocols in the data abstraction tool.

Demographic information and provider characteristics collected included maternal education (high school graduate or less, some college, 4 years of college or more), marital status (single or married/partnered), insurance coverage (public or commercial/employer), and type of prenatal care provider (academic or private provider). Medical risk factors associated with pregnancy complications included maternal age, parity, body mass index (BMI) at delivery, gestational age, birth weight, and trimester of first entry into prenatal care. Any hypertension, any diabetes, multiple gestation, and prior cesarean delivery were collected as dichotomous (yes/no) variables. Several obstetric characteristics were collected including onset of labor (spontaneous, pre-labor, or induction). A cesarean done prior to the onset of labor was referred to as a “scheduled” cesarean delivery.

To assess the quality of the manual abstraction, inter-rater reliability was calculated with κ-statistics and inter-class correlations depending on whether the variable was categorial or continuous. This was done for the first 45 charts (7.2%) that were independently abstracted by both collectors. The level of agreement was found to be almost perfect (kappa = 0.81–1.00) for every variable except gestational hypertension, which had substantial agreement (kappa = 0.78).

All statistical analyses were conducted using STATA (StataCorp LLC, College Station, TX, USA). Descriptive statistics were used to characterize the sample and chi square tests to assess for differences in the sample characteristics for Micronesian and White women. We also described with proportions the primary indications provided for the full sample and primary indications by race/ethnicity. A chi square test was used to assess the statistical significance of differences in indication by race/ethnicity. We define statistically significant results as those with a *p*-value (*P*) of less than or equal to 0.05.

Multivariable logistic regression models provided estimates of association between race/ethnicity and the indication of cesarean categorized as: (1) subjective/objective indications, (2) NRFHT, and (3) arrest of labor. Our models were adjusted for the hypothesized confounders of maternal age, parity (in four categories), diabetes (any condition), hypertension (any, excluding pre-eclampsia), and birth weight. These covariates, with the exception of parity, were also included in a previous study conducted in Hawai‘i documenting a significantly higher rate of cesarean delivery among Micronesian compared to White women [14]. Parity was included here because the previous work [11] noted its omission as a limitation in their analyses. Diabetes and hypertension were treated as confounders based on previous work [2,14], but may in fact be on the causal pathway between race and indication for cesarean delivery. Thus, we may overadjust (i.e., underestimate any associations) with these models. The model fit was tested using a Hosmer and Lemeshow goodness-of-fit test.

Subsets of patients who had a primary cesarean delivery, who experienced spontaneous onset of labor, and singleton births were selected to check if results obtained analyzing the full sample were replicated in the restricted samples. The subset analyses were conducted because each condition (primary cesarean, spontaneous labor, and singleton) excludes or accounts for certain risks. This provided a way to account for the complexity of cesarean while reducing the risk of overadjustment in the full model. For example, an analysis restricted to singletons eliminates the influence of multiple gestation, which can have implications on the birth weight and gestational age. Looking only at spontaneous deliveries accounts for the fact that women cannot experience certain indications (e.g., arrest of labor) without having progressed to labor.

Finally, we ran sensitivity analyses in which we included maternal BMI, education, and trimester of first prenatal care visit (see Supplementary Materials Table S1). We considered these three variables to be on the causal pathway between maternal race/ethnicity and indication for cesarean delivery. For example, Micronesian women may be more likely to encounter obstacles to accessing care and hence be more likely to receive late prenatal care relative to other groups. This could increase the likelihood of experiencing a cesarean delivery due a lower threshold for risk among providers who have relatively little information about the patient’s medical history and risk factors [18]. While not confounders, inclusion of these variables in a sensitivity analysis provides insights into the robustness of the associations observed between race/ethnicity and subjective indication for cesarean delivery. However, this supplemental table should be interpreted with caution because the model is overadjusted, and there is likely collinearity between variables such as maternal education and initiation of prenatal care.

## 3. Results

A comparison of demographic and provider characteristics revealed significant differences between the White and Micronesian groups, with White women more likely to have private insurance (82% vs 13%) and higher levels of education (≥4 years of college 58% vs 4%). Maternal and obstetric characteristics also largely differed between groups (Table 1). Some variables exposed divergent patterns between groups. For example, White women in this sample were more likely to see a prenatal care provider in the first trimester (73%) compared to Micronesian women (27%). Twenty-nine percent of Micronesian women-initiated care in the third trimester compared to 8% of White women.

**Figure 1:**
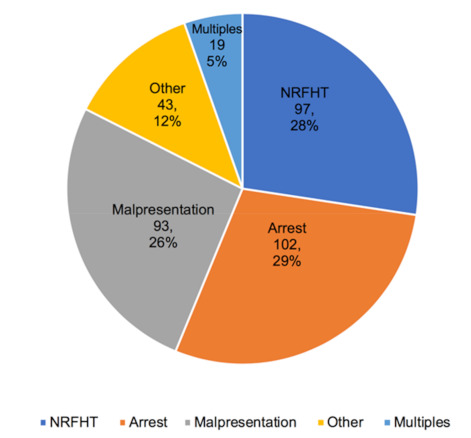
Major indications for primary cesarean delivery (N = 354).

**Figure 2:**
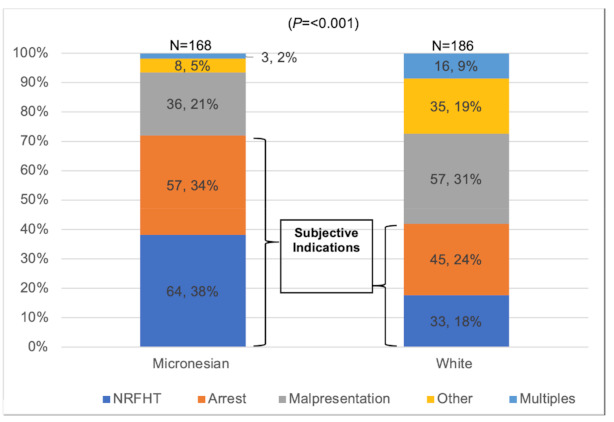
Major indications for primary cesarean delivery by race/ethnicity (N = 354).

The most common indications for cesarean in the full sample (N = 620) were repeat cesarean (30%), followed by arrest disorders (20%), NRFHT (19%), and malpresentation (19%). Figure 1 shows the distribution of indications for only primary cesarean deliveries (N = 354). The recorded indications for cesarean delivery were significantly different for White and Micronesian women in our sample. Micronesian women experienced a higher percentage of cesarean delivery for the more subjective indications—NRFHT and arrest of labor disorders—in both the full sample and among primary cesarean deliveries. Figure 2 shows the indications for primary cesarean delivery by race/ethnicity.

Table 2 shows the results from the multivariate regression models. The analyses reveal that Micronesian women had significantly greater odds of having a cesarean delivery due to subjective indications (NRFHT or arrest of labor) compared to objective indications (aOR: 4.17; CI: 2.52–6.88; *P* < 0.001) after adjusting for age, parity, any diabetes, any hypertension, and newborn birth weight. When looking at each subjective indication independently, we found Micronesian women had nearly three times the likelihood of having a cesarean for NRFHT compared to White women (aOR: 2.91; CI: 1.74–4.90; *P* < 0.001). The adjusted odds of having a cesarean due to an arrest disorder were also higher for Micronesian compared to White women (aOR: 1.85; CI: 1.07–3.20; *P* = 0.028), but the magnitude of association was less than for NRFHT.

The results of the sensitivity analyses corroborated previous findings (see Table 3); the same patterns were seen for subjective indications overall and for each one individually. Even in a model overadjusted for variables likely on the causal pathway between race/ethnicity and cesarean delivery (see Table S1), there was a statistically significant association between subjective indication and Micronesian race (aOR: 3.09; CI: 1.63–5.85; *P* = 0.001).

## 4. Discussion

This study sought to understand possible factors underlying the higher cesarean rates for Micronesian compared to White women in Hawai‘i. The findings supported our hypothesis that Micronesian women would be more likely to have had a cesarean delivery due to a more subjective indication (defined as NRFHT or arrest of labor) compared to White women. Our findings were statistically significant even after adjusting for numerous risk factors. The difference was largely driven by the relationship between race/ethnicity and NRFHT, which was consistently demonstrated in examinations of subsets of deliveries including primary cesarean delivery only, singleton births only, and deliveries that only involved spontaneous labor.

Our findings are congruent with other research examining racial/ethnic disparities in cesarean delivery [2,3,13,28]. Washington et al. (2012) found that Black women in their sample were twice as likely to have a cesarean delivery for NRFHT compared to White women after adjusting for confounders and that Black, Latina, and Asian women all had higher odds of having a cesarean due to “failure to progress” (similar to the arrest of labor category in our study) compared to White women [2]. Another study of 750 women at low risk for cesarean delivery found that Black women were at higher risk of cesarean delivery for “fetal distress” (98.3% of indications in that category were NRFHT or intrapartum fetal distress) compared to White women in a model adjusted for BMI, maternal age, and neonate size [13]. Our study adds to the existing literature by examining a racial/ethnic group that is understudied and whose outcomes are often masked due to data aggregation with other racial/ethnic groups (e.g., Native Hawaiian or Asians). When considered alongside other similar literature, our work demonstrates a pattern in which racial/ethnic minority groups in the U.S. not only experience higher rates of cesarean delivery, but also a greater proportion of these are for more subjective indications that may be prone to implicit and explicit provider biases [3]. This work further built on previous literature by also including variables that were not consistently captured in previous research that examines disparities in cesarean delivery by race/ethnicity, such as BMI, parity, trimester of entry into prenatal care, and induction of labor.

While not definitive, our findings considered within the broader literature on the subject raise concerns that provider biases may impact clinical decision-making and recommendations on cesarean delivery for racial/ethnic minorities given the recognized subjectivity and variation in the interpretation of fetal heart tracings and arrest disorders. In the face of unexplained racial/ethnic disparities in cesarean delivery, a critical examination of clinical decision-making, including considerations of implicit bias, is important. Research into the relationship between implicit bias and health/healthcare outcomes is expanding. A recent review found that provider–patient communication and, in some cases, treatment decisions are associated with measures of implicit racial bias [29]. Studies that investigate the role of implicit bias within obstetric decision-making are limited, but there is increasing interest in understanding and addressing the role of racial bias in obstetrics in the U.S., particularly in the face of increasing pregnancy-related mortality [7,8,30]. While the focus of much of the research in the U.S. has examined differences between Black and White populations, the rationale could be extended to Micronesian communities in Hawai‘i and as well as migrant or immigrant communities in other settings that also encounter explicit discrimination, prejudice and implicit bias.

We would add that investigations into physician and patient factors impacting diagnosis of NRFHT and arrest disorder may improve understanding of factors affecting differences by race/ethnicity. In addition, an important next step would be to examine the relationship between indication for cesarean delivery and racial/ethnic identity in a larger data set (with vaginal, operative, and cesarean deliveries from multiple institutions).

### Strengths and Limitations

There are limitations of this research that should be noted. First, the study data were from one hospital. This institution handles nearly half of the deliveries in O‘ahu, and the sample is likely representative of Micronesian women in the state; however, the White women in the sample may represent higher risk pregnancies compared to the broader population in Hawai‘i. Second, data on patient preference for vaginal vs. cesarean delivery were not collected. Patient preference could have implications for the risk and, possibly, the indication for cesarean delivery if, for example, Micronesian women are more likely to request a vaginal delivery compared to White women in the face of a risk factor unmeasured in this study (e.g., history of shoulder dystocia). Alternatively, if more Micronesian women compared to White women prefer to attempt a version in the case of breech presentation, they may be less likely to experience a cesarean delivery for malpresentation. However, it remains unclear if preference could account for the substantial differences observed here or the higher frequency of cesarean delivery observed for Micronesian women in statewide data.

This research study had several strengths. First, it addressed an understudied population and expanded on existing research (conducted within the same state) to gain in-depth knowledge of observed racial/ethnic disparities. Second, it was hypothesis-driven. Thirdly, the sample selection was randomized and followed a detailed and multistep data collection protocol.

## 5. Conclusions

Our results overall suggest that Micronesian patients in this sample were more likely to experience a cesarean delivery for indications that involve a greater level of clinician interpretation and discretion than White women. Although the Micronesian population is small relative to other racial/ethnic groups within the U.S., the methods and results presented here may be helpful in understanding unexplained disparities in cesarean delivery among other understudied racial/ethnic minority groups in the U.S. and other nations.

## Figures and Tables

**Table 1:** Maternal and obstetric factors by race/ethnicity (N = 620).

Characteristic (Miss.)	Micronesian		White		Full Sample		*P* ^a^
	N	%	N	%	N	%	
Maternal age (0)							
<20	39	12.04	6	2.03	45	7.26	<0.001
20–25	86	26.54	34	11.49	120	19.35	
26–30	97	29.94	76	25.68	173	27.90	
31–35	67	20.68	93	31.42	160	25.81	
36–40	30	9.26	67	22.64	97	15.65	
41 and over	5	1.54	20	6.76	25	4.03	
Total	324	100	296	100	620	100	
Parity (1)							
0	110	34.06	150	50.68	260	42.00	<0.001
1	71	21.98	81	27.36	152	24.56	
2	65	20.12	37	12.50	102	16.48	
3	42	13.00	16	5.41	58	9.37	
4 or more	35	10.84	12	4.05	47	7.59	
Total	323	100	296	100	619	100	
Any hypertension (0)							
Yes	35	10.80	35	11.82	70	11.29	0.688
No	289	89.20	261	88.18	550	88.71	
Total	324	100	296	100	620	100	
Any diabetes (0)							
Yes	51	15.74	20	6.76	71	11.45	<0.001
No	273	84.26	276	93.24	549	88.55	
Total	324	100	296	100	620	100	
Multiple gestation (0)							
Multiple	14	4.32	56	18.92	70	11.29	<0.001
Singleton	310	95.68	240	81.08	550	88.71	
Total	324	100	296	100	620	100	
Prior cesarean (0)							
Yes	156	48.15	110	37.16	266	42.90	0.006
No	168	51.85	186	62.84	354	57.10	
Total	324	100	296	100	620	100	
Onset of labor (2)							
Spontaneous	162	50.00	94	31.97	256	41.42	<0.001
Pre-labor	110	33.95	156	53.06	266	43.04	
Induction	52	16.05	44	14.97	96	15.53	
Total	324	100	294	100	618	99.99	
Birth weight (0)							
<2500g	56	17.28	67	22.64	123	19.84	0.037
2500–3499g	156	48.15	150	50.68	306	49.35	
3500–3999g	75	23.15	62	20.95	137	22.10	
≥4000g	37	11.42	17	5.74	54	8.71	
Total	324	100	296	100	620	100	

Abbreviations: Miss., missing. ^a^ Chi square test for independence.

**Table 2:** Adjusted ^a^ odds of cesarean delivery for subjective indication, NRFHT, and arrest of labor (N = 619).

Variable		Subjective			NRFHT			Arrest		
		aOR	(95% CI)	*P*	aOR	(95% CI)	*P*	aOR	(95% CI)	*P*
Race/ethnicity	Micronesian	4.17	2.52–6.88	<0.001	2.91	1.74–4.90	<0.001	1.85	1.07–3.20	0.028
	White	ref			ref			ref		
Age	continuous	0.96	0.93–1.00	0.026	0.97	0.93–1.00	0.081	0.99	0.95–1.03	0.632
Parity	0	9.57	4.97–18.44	<0.001	3.80	1.83–7.89	<0.001	4.99	2.33–10.68	<0.001
	1	1.24	0.64–2.38	0.525	1.68	0.77–3.69	0.195	0.76	0.32–1.79	0.530
	2	0.45	0.21–0.97	0.040	0.86	0.35–2.12	0.738	0.25	0.08–0.83	0.023
	≥3	ref			ref			ref		
Diabetes	Yes	1.39	0.73–2.62	0.318	0.93	0.46–1.87	0.834	1.62	0.75–3.49	0.218
	No	ref			ref			ref		
Hypertension	Yes	1.51	0.81–2.79	0.193	0.84	0.42–1.66	0.612	2.21	1.11–4.39	0.024
	No	ref			ref			ref		
Birth weight	continuous by 100 g	1.08	1.05–1.11	<0.001	0.99	0.96–1.01	0.338	1.157	1.11–1.21	<0.001

**^a^** Odds ratios are calculated by multivariable logistic regression and are adjusted for all other covariates in the table.

**Table 3:** Adjusted ^a^ odds of cesarean delivery by race/ethnicity for subjective indications for three subsets.

Subset	Race/Ethnicity ^b^	Subjective			NRFHT			Arrest		
		aOR	(95% CI)	*P*	aOR	(95% CI)	*P*	aOR	(95% CI)	*P*
Primary Cesarean Only (N = 353)										
	Micronesian	3.36	1.83–6.17	<0.001	2.33	1.29–4.19	0.005	1.46	0.78–2.72	0.235
Spontaneous Labor Only (N = 256)										
	Micronesian	6.01	2.58–13.99	<0.001	3.05	1.39–6.67	0.005	1.91	0.86–4.24	0.112
Singleton Births Only (N = 549)										
	Micronesian	4.06	2.39–6.87	<0.001	2.8	1.64–4.78	<0.001	1.75	1.01–3.03	0.048

**^a^** Odds ratios are calculated by multivariable logistic regression and are adjusted for maternal age, parity, diabetes, hypertension, and birth weight. **^b^** White is the reference group.

## Data Availability

Restrictions apply to the availability of these data. Data was obtained from Hawai‘i Pacific Health and are available from the authors with permission of Hawai‘i Pacific Health.

## Supplementary Materials

The following are available online at https://www.mdpi.com/2227-9032/9/2/159/s1, Table S1: Overadjusted multivariate model examining the association between race/ethnicity and subjective indication for cesarean section (N = 619).
