# Supplementary material for: A Cross-Sectional Study Examining Differences in Indication for Cesarean Delivery by Race/Ethnicity
Source: Healthcare (Basel). 2021 Feb 3;9(2):159. doi: 10.3390/healthcare9020159 (PMC7913232; doi:10.3390/healthcare9020159)
Supplement: Supplementary file 1 [file healthcare-09-00159-s001.pdf]

## Supplementary File

**Table S1. Overadjusted multivariate model examining the association between race/ethnicity and subjective indication for cesarean delivery (N=619)**

| | Subjective indication | | |
|---|---|---|---|
| **Variable** | **aOR[a]** | **95% CI** | ***P*** |
| **Race/ethnicity** | | | |
| **Micronesian** | 3.091 | 1.633-5.850 | 0.001 |
| **White** | *ref* | | |
| **Age** | | | |
| **(continuous)** | 0.994 | 0.955-1.036 | 0.788 |
| **Parity** | | | |
| **0** | 18.532 | 8.338-41.189 | <0.001 |
| **1** | 1.801 | 0.842-3.852 | 0.129 |
| **2** | 0.518 | 0.218-1.233 | 0.137 |
| **≥3** | *ref* | | |
| **Diabetes** | | | |
| **Yes** | 1.488 | 0.754-2.936 | 0.252 |
| **No** | *ref* | | |
| **Hypertension** | | | |
| **Yes** | 1.603 | 0.781-3.290 | 0.198 |
| **No** | *ref* | | |
| **Birth weight** | | | |
| **By 100g (continuous)** | 1.094 | 1.056-1.133 | <0.001 |
| **Maternal BMI** | | | |
| **>35 (obese)** | 1.011 | 0.976-1.047 | 0.531 |
| **<35** | *ref* | | |
| **Initiation of prenatal care** | | | |
| **1st trimester** | *ref* | | |
| **2nd trimester** | 0.913 | 0.532-1.567 | 0.742 |
| **3rd trimester** | 1.495 | 0.789-2.831 | 0.217 |
| **Maternal education** | | | |
| **High school graduate or less** | *ref* | | |
| **Some college** | 0.454 | 0.256-0.803 | 0.007 |
| **≥4 years of college** | 0.300 | 0.129-0.698 | 0.005 |

Abbreviation: aOR: adjusted odds ratio

[a] Odds ratios calculated by multivariable logistic regression and adjusted for each of the other covariates listed in the table
